# Comparison of 3-Factor Prothrombin Complex Concentrate and Low-Dose Recombinant Factor VIIa for Warfarin Reversal

**DOI:** 10.1186/1749-7922-9-27

**Published:** 2014-04-15

**Authors:** Scott A Chapman, Eric D Irwin, Nada M Abou-Karam, Nichole M Rupnow, Katherine E Hutson, Jeffrey Vespa, Robert M Roach

**Affiliations:** 1 Department of Experimental and Clinical Pharmacology, University of Minnesota College of Pharmacy, 7-115E Weaver Densford Hall 308 Harvard Street S.E, Minneapolis, MN 55455, USA; 2 Department of Trauma, North Memorial Medical Center, Robbinsdale, MN, USA; 3 University of Minnesota College of Pharmacy, 5-130 Weaver-Densford Hall, 308 Harvard Street SE, Minneapolis, MN 55455, USA; 4 Department of Pharmacy Services, North Memorial Medical Center, Minneapolis, USA; 5 Department of Pharmaceutical Care and Health Systems, University of Minnesota College of Pharmacy, Minneapolis, USA; 6 Department of Emergency Medicine, North Memorial Medical Center, Minneapolis, USA

**Keywords:** Anticoagulation, Hemorrhage, Trauma, Prothrombin complex concentrate, Recombinant factor VIIa, Warfarin

## Abstract

**Introduction:**

Prothrombin complex concentrate (PCC) and recombinant Factor VIIa (rFVIIa) have been used for emergent reversal of warfarin anticoagulation. Few clinical studies have compared these agents in warfarin reversal. We compared warfarin reversal in patients who received either 3 factor PCC (PCC3) or low-dose rFVIIa (LDrFVIIa) for reversal of warfarin anticoagulation.

**Methods:**

Data were collected from medical charts of patients who received at least one dose of PCC3 (20 units/kg) or LDrFVIIa (1000 or 1200 mcg) for emergent warfarin reversal from August 2007 to October 2011. The primary end-points were achievement of an INR 1.5 or less for efficacy and thromboembolic events for safety.

**Results:**

Seventy-four PCC3 and 32 LDrFVIIa patients were analyzed. Baseline demographics, reason for warfarin reversal, and initial INR were equivalent. There was no difference in the use of vitamin K or fresh frozen plasma. More LDrFVIIa patients achieved an INR of 1.5 or less (71.9% vs. 33.8%, p =0.001). The follow-up INR was lower after LDrFVIIa (1.25 vs. 1.75, p < 0.05) and the percent change in INR was larger after LDrFVIIa (54.1% vs. 38.8%, p = 0.002). There was no difference in the number of thromboembolic events (2 LDrFVIIa vs. 5 PCC3, p = 1.00), mortality, length of hospital stay, or cost.

**Conclusions:**

Based on achieving a goal INR of 1.5 or less, LDrFVIIa was more likely than PCC3 to reverse warfarin anticoagulation. Thromboembolic events were equivalent in patients receiving PCC3 and LDrFVIIa.

## Introduction

Bleeding complications continue to be an important risk of warfarin anticoagulation.

**Figure 1:**
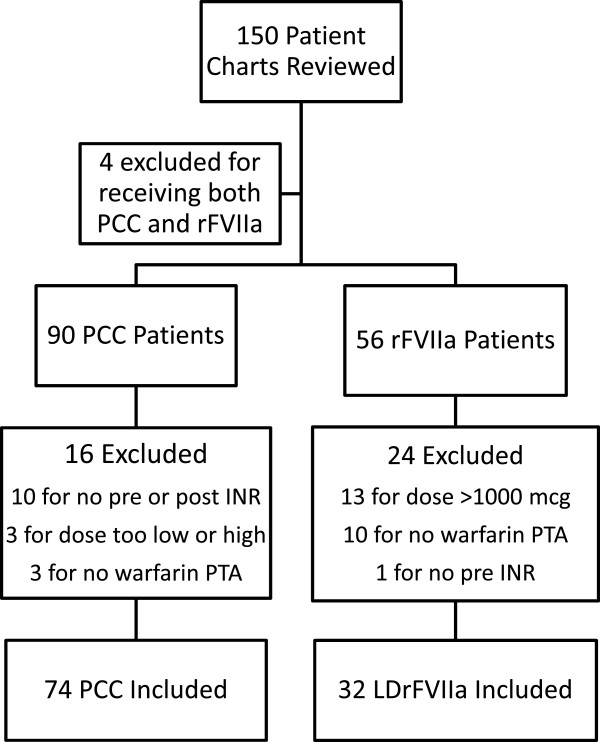
**Subject selection.** PCC3, 3 factor prothrombin complex concentrate; LDrFVIIa, low dose recombinant factor VII activated.

Despite this risk, warfarin continues to be a widely used anticoagulant for outpatient management of patients who have suffered a deep vein thrombosis with or without pulmonary embolism, or who require prophylaxis against a thromboembolic event associated with atrial fibrillation or prosthetic valves. Furthermore, as the population continues to age, the number of patients receiving warfarin is increasing and this correlates with a rise in the incidence of complications associated with warfarin anticoagulation. This ultimately results in an increase in risk for bleeding and associated morbidity and mortality for patients. In a pooled analysis of 3665 patients receiving warfarin anticoagulation (goal international normalized ratio [INR] 2.0- 3.0) for nonvalvular atrial fibrillation in the SPORTIF III and V trials, the annual incidence of major bleeding and associated mortality was 2.68% and 8.09%, and the incidence of intracerebral bleeding and associated mortality was 0.19% and 45.4% [1].

Patients who suffer severe or life-threatening bleeding complications during warfarin anticoagulation require rapid normalization of their coagulation status in an attempt to minimize bleeding and the associated morbidity. Traditionally, this is achieved by transfusion of fresh frozen plasma (FFP) to provide functional coagulation factors and administering vitamin K. Disadvantages of FFP includes the large volume of fluid required, the time required to thaw, the time need for blood group matching, and the risk for transfusion reactions, transmission of infections and transfusion related lung injury. For intravenous vitamin K there is a small risk of anaphylaxis (3 per 10,000 patients) [2]. Finally, both strategies require significant time to normalize the patient’s INR (median time > 8–32 hours for FFP and > 24 hours for vitamin K) [3-9].

The coagulation factors prothrombin complex concentrate (PCC) and recombinant factor VII activated (rFVIIa) have both been used for the reversal of warfarin anticoagulation. Both can be administered more quickly and can provide more rapid reversal of warfarin anticoagulation as defined by normalization of the INR [10-14]. The doses of PCC and rFVIIa administered in these reports has varied widely and thus the optimal dose for reversal of warfarin anticoagulation with these products is unknown. Additionally, there is little information about potential differences in the efficacy and safety of rFVIIa when compared with PCC.

There is limited data in the literature reporting a comparison of PCC and rFVIIa for warfarin anticoagulation reversal [14]. Our institution uses both a 3 factor PCC (PCC3) weight based doses at 20 units/kg regardless of INR and low dose rFVIIa (LDrFVIIa) 1000 mcg or 1200 mcg for serious and life-threatening bleeding in patients anticoagulated with warfarin. To evaluate these therapies, we reviewed the charts of patients who required emergent reversal of warfarin anticoagulation and who received either PCC as a 3 factor product (PCC3) or LDrFVIIa to compare the safety and efficacy of these coagulation factor products. Our hypothesis was that PCC3 and LDrFVIIa are equally effective and safe for warfarin anticoagulation reversal.

## Methods

Institutional review board approval was obtained and a retrospective chart review was conducted at North Memorial Medical Center, an American College of Surgeons verified level 1 trauma center. The electronic medical record database was searched to identify all patients who received either PCC or rFVIIa from August 29th, 2007 to October 10th, 2011. A review of the electronic medical record of those patients was conducted to identify patients who met the following inclusion criteria: Clear documentation of warfarin usage prior to admission, a need for emergent reversal of warfarin anticoagulation and a pre-reversal INR of 1.6 or greater, received either prothrombin complex concentrate (PCC3, 20 units/kg rounded to nearest 500 units) or low-dose recombinant Factor VIIa (LDrFVIIa, 1000 or 1200 mcg), and at least one INR obtained pre and one INR obtained post coagulation factor administration. Fresh frozen plasma and vitamin K were administered at provider discretion. Patients were excluded if they had no pre or post coagulation factor INR, a pre-reversal INR of 1.5 or less, received both PCC3 and LDrFVIIa, received more than one PCC3 or rFVIIa dose before follow-up INR, or received any single rFVIIa dose greater than 1200 mcg. The PCC3 product used was Profilnine® SD (Grifols Biologicals Inc., Los Angeles, CA) and the rFVIIa product was NovoSeven® or NovoSeven RT® (Novo Nordisk Inc., Princeton, NJ).

The following data were collected: 1) Demographic: age, gender, indication for warfarin, and indication for reversal; 2) Coagulation parameters: INR pre and post administration of either PCC3 or LDrFVIIa, change in INR (absolute and percent change), achievement at INR of 1.5 or less, and time to reach INR 1.5 or less; 3) Reversal agent administration and cost: PCC3, LDrFVIIa, FFP, vitamin K; 4) Patient outcome: Mortality, hospital length of stay, survivor length of stay, and incidence and type of thromboembolic event. There was no systematic schedule for INR monitoring after the administration of reversal agents, and repeat doses of coagulation factors were administered at the treating provider’s discretion based on the follow-up INR after the first dose. There was no systematic screening for thromboembolic events; patients were assessed for any potential thromboembolic complications as was deemed clinically appropriate.

Data were compared between the two groups to determine if differences were statistically significant for the above-mentioned demographic, coagulation, and outcome parameters, with the primary efficacy end-point being achievement of goal INR less than or equal to 1.5, and the primary safety end-point being number of thromboembolic events. Statistical tests utilized include the Wilcoxon Rank Sum test to compare continuous data, reported as median [IQR], and Chi Square or Fisher exact test for categorical data, reported as n, %. A p value less than or equal to 0.05 was considered statistically significant.

## Results

Based on inclusion and exclusion criteria, 74 PCC3 patients and 32 LDrFVIIa patients were included in the final analysis (Figure 1). There were no significant differences between the groups with regards to age, gender, or indication for anticoagulation with warfarin (Table 1). There was also no difference in the indication for emergent reversal (Table 2), except for more patients who presented with subdural hematoma received LDrFVIIa. The groups were similar with regards to the percentage of patients receiving vitamin K (77.0% PCC3 vs. 68.8% LDrFVIIa, p = 0.37) or FFP (66.2% PCC3 vs. 65.6% LDrFVIIa p = 0.95), and the number FFP units administered (2[0-4] PCC3 vs. 2[0-4] LDrFVIIa, p = 0.75) (Table 3). The initial dose of PCC3 was 1540[1429-1978] units or 19.9 [18.6-20.8] units/kg, and the dose of LDrFVIIa was 1000[1000-1000] mcg or 11.5 [10.1-15.0] mcg/kg. Table 4 details the INR response comparing the two coagulation factors. Baseline INRs were equivalent for the two groups prior to the first dose of either PCC3 or LDrFVIIa (3.1[2.3-4.1] PCC3 vs. 2.8[2.2-3.6] LDrFVIIa, p = 0.52). After one dose of coagulation factor, 71.9% of patients in the LDrFVIIa group achieved goal INR of 1.5 or less compared to 33.8% in the PCC3 group (p = 0.001). The time between pre and post coagulation factor INRs was similar (3:53[2:32-7:17]) in PCC3 group and 4:30[2:21-6:25] in LDrFVIIa group, p = 0.78). The percent change in INR was higher after administration of LDrFVIIa compared to PCC3 (54.1% [47.3%-62.7%] for the LDrFVIIa group vs. 38.8% [30.7%-56.0%] for the PCC3 group, p = 0.002).

**Table 1:** Baseline demographic characteristics of the study patients

**Characteristics**	**PCC3 (n = 74)**	**LD rFVIIa (n = 32)**	***p***
Demographics			
Age (years)*	73 [62.3-81.0]	67 [59.5-79.3]	0.32
M:F	43:31	22:10	0.39
Indication for Warfarin, n*,#, **, ***			
Atrial arrhythmia	44	19	0.99
Cardiomyopathy	2	1	1.00
Valve replacement	11	7	0.38
Ischemic CVA	2	2	0.58
DVT/PE			
Treatment*#	18	6	0.53
Prophylaxis	11	3	0.55
Portal vein thrombosis	0	1	0.30
Hyperhomocysteinemia	1	0	1.00
Lupus Anticoagulant	1	0	1.00
Syndrome			
Unknown	1	0	1.00

*2 with Protein S deficiency # 2 with Anticardiolipin Syndrome.

**5 with 2 indications ***5 with 2 indications.

*Data reported as median [IQR].

PCC3, 3 factor prothrombin complex concentrate; LDrFVIIa, low dose recombinant factor VII activated; CVA, cerebral vascular accident; DVT, deep vein thrombosis; PE, pulmonary embolism.

**Table 2:** Indication for warfarin anticoagulation reversal

**Characteristics**		**PCC3 (n = 74)**	**LD rFVIIa (n = 32)**	***p***
Neuro, n*		39	23	0.07
CH		19	9	0.79
SDH		7	9	0.014
SAH		6	2	1.00
SCI		1	2	0.22
TBI		6	1	0.67
Craniotomy		0	1	0.30
Abdominal		11	3	0.55
Intraperitoneal Hem.		2	0	1.00
Retroper. hematoma		1	0	1.00
GIB		2	1	1.00
Perf. Viscous/		0	1	0.30
peritonitis				
Pneumoperitoneum		1	0	1.00
Incarcerated hernia		2	1	1.00
Acute abdomen		1	0	1.00
Diverticulitis		1	0	1.00
Colonic perforation		1	0	1.00
Other	25	8	0.37	
Orthopedic	2	3	0.16	
Fall w/external inj.	0	1	0.30	
	Multiple trauma	0	1	0.30
	Pulmonary contusion	1	0	1.00
Chest wall trauma	1	0	1.00	
	Pacemaker placement	2	0	1.00
	Emergent surgery	4	1	1.00
	Ruptured iliac	1	0	1.00
	Artery aneurysm			
	Pseudoaneurysm	1	0	1.00
	CFA			
	Hematoma	3	0	1.00
	Pneumothorax	2	0	1.00
	Posthemorrhagic	1	0	1.00
	Hydrocephalus			
	Epistaxis	0	1	0.30
	INR > 8	6	0	0.18
	Unknown	1	0	1.00

*1 with more than 1 indication.

PCC3, 3 factor prothrombin complex concentrate; LDrFVIIa, low dose recombinant factor VII activated; ICH, intracranial hemorrhage, SDH, subdural hematoma, SAH, subarachnoid hemorrhage, SCI, spinal cord injury, TBI, traumatic brain injury, GIB, gastrointestinal bleed, CVA, cerebral vascular accident; DVT, deep vein thrombosis; PE, pulmonary embolism.

**Table 3:** Warfarin anticoagulation reversal agents prescribed

	**PCC3 (n = 74)**	**LD rFVIIa (n = 32)**	***p***
*Initial coagulation factor dose*			
Total Dose (units)*	1540 [1429-1978]	1000 [1000-1000]	NA
Weight-based Dose (units/kg)*	19.9 [18.6-20.8]	11.5 [10.1-15.0]	NA
*Other reversal agents administered*			
Vit K, n (%)	57 (77.0%)	22 (68.8%)	0.37
FFP, n (%)	49 (66.2%)	21 (65.6%)	0.95
FFP units*	2 [0-4]	2 [0-4]	0.75
*Total cost for reversal agents*:			
Coagulation factor (USD)*:	1116.50 [963-1718]	1230 [1170-1360]	0.26
FFP(USD)*:	393 [0-496]	393 [0-496]	0.65
Total(USD)*:	1526 [1299-2047]	1609.50 [1360-1756]	<0.05

*Data as median [IQR].

PCC3, 3 factor prothrombin complex concentrate; LDrFVIIa, low dose recombinant factor VII activated; kg, kilograms; FFP, fresh frozen plasma; vit K, vitamin K, USD, United States Dollars).

**Table 4:** INR response after the first dose of PCC3 or LDrFVIIa

	**PCC3 (n = 74)**	**LD rFVIIa (n = 32)**	***p***
INR baseline*:	3.1 [2.3-4.1]	2.8 [2.2-3.6]	0.52
INR post coagulation factor*:	1.75 [1.5-2.0]	1.25 [1.0-1.25]	<0.05
INR Δ*:	1.2 [0.7-2.2]	1.5 [1.2-2.0]	0.14
% Δ INR*:	38.8% [30.7%-56.0%]	54.1% [47.3%-62.7%]	0.002
n (%) ≤ 1.5:	25 (33.8%)	23 (71.9%)	0.001
Time (h:mm)*:	3:53 [2:32-7:17]	4:30 [2:21-6:25]	0.78

*Data as median [IQR].

PCC3, 3 factor Prothrombin Complex Concentrate; LDrFVIIa, low dose recombinant factor VII activated; INR, International Normalized Ratio.

Five thromboembolic events occurred in the PCC3 group compared to 2 events in the LDrFVIIa group (Table 5, p = 1.00). Deep vein thrombosis (DVT) occurred in 2 patients in each group. In the PCC3 group, one patient was found to have 4 upper extremity DVTs 7 days after PCC3 administration, and the other was found to have a superior femoral vein DVT 5 days after PCC3 administration. In the LDrFVIIa group, one patient had a lower extremity DVT 11 days after LDrFVIIa administration, and the other was found to have a left upper extremity non- occlusive DVT 7 days post-LDrFVIIa. All DVTs diagnosed by duplex ultrasonography. Three PCC3 patients experienced an additional thromboembolic complication during their hospitalization: right internal jugular vein thrombus 15 days post-PCC3 (central line present), MRI-confirmed cerebrovascular accident (CVA) with multiple infarcts 2 days post-PCC3, and chest tube clots 1 day post-PCC3 (this patient may have also had a CVA which could have contributed to death, although this was not confirmed with imaging).

**Table 5:** Patient outcomes

	**PCC3 (n = 74)**	**LD rFVIIa (n = 32)**	***p***
Mortality, n (%)	22 (29.7%)	6 (18.8%)	0.34
LOS all pts (d)*	8.0 [4-11]	7.5 [5-13]	0.43
LOS survivors (d)*	8.0 [4-11]	9.5 [6-13]	0.15
Thromboembolic events	5	2	1.00
DVT	2	2	
IJ thrombus	1	0	
Multiple CVA’s	1	0	
Chest tube clots	1	0	
(and possible unconfirmed CVA)			

*Data as median [IQR].

PCC3, 3 factor prothrombin complex concentrate; LDrFVIIa, low dose recombinant factor VII activated; LOS, length of stay; DVT, deep vein thrombosis, IJ, internal jugular; CVA, cerebral vascular accident.

There was no difference in mortality (29.7% PCC3 vs. 18.8% LDrFVIIa, p = 0.34), overall length of hospital stay [PCC3 group 8.0 [4-11] days vs. LDrFVIIa group 7.5 [5-13] days (p = 0.43)] or length of stay of survivors [PCC3 group 8.0 [4-11] days vs. LDrFVIIa group 9.5 [6-13], p = 0.15]. Coagulation factor cost (USD) was not different (1116.50 [963-1718] in the PCC3 group, and 1230[1170-1360] in the LDrFVIIa group, p = 0.26) and FFP cost (USD) was similar between the two groups (393[0-496] in the PCC3 group and 393[0-496] in the LDrFVIIa group, p = 0.70). However, when combined, the overall cost for FFP and coagulation factor was higher in the PCC3 group (1526 [1299-2047] PCC3 vs. 1609.50 [1360-1756] LDrFVIIa, p < 0.05).

## Discussion

Administration of PCC or rFVIIa for the reversal of warfarin anticoagulation in emergent or life-threatening bleeding has been reported in several publications, yet there is uncertainty as to their safety or efficacy, and the appropriate doses of these agents to administer. Our findings indicate that LDrFVIIa (1000 or 1200 mcg) is more effective at reversing the INR compared to PCC3 (20 units/kg) as evident by more patients achieving an INR of 1.5 or less. Furthermore, only one patient receiving LDrFVIIa required a second dose for additional warfarin reversal, compared to 16 PCC3 patients who received a second dose, all of these due to failure of the first dose to effectively reverse the INR to 1.5 or less. There was no difference in mortality or thromboembolic complications, although the small sample size makes this difficult to interpret. Further, no association can be made from this data as to whether the thromoboembolic events were the result of the coagulation factor administered independent of other existing risk factors for thromboembolic events.

Prothrombin complex concentrate products are derived from purified pooled human plasma. All PCC products contain factors II, IX, and X along with variable amounts of factor VII. Some PCC products, referred to as 4 factor PCC, contain larger amounts of factor VII (36–100 I.U. per 100 I.U. factor IX) compared to PCC3 products, that contain relatively low amounts of factor VII (0–25 I.U. per 100 I.U. factor IX) [11]. Both PCC3 products (dosed at 12–50 units/kg) and 4 factor PCC products (dosed at 7–50 units/kg) have been reported to provide rapid reversal of the INR [11]. Two PCC products available in the United States (Profilnine® SD and Bebulin® VH) are PCC3 products. Give the absence of a standardized dosing regimen at the time of this work and the wide range of doses of PCC reported in the literature, we chose 20 units/kg as an initial PCC dose with recommendations to repeat the INR post-PCC3 administration. A 4 factor PCC product available in Europe has completed clinical trials and has recently been approved by the FDA (Kcentra®) for warfarin reversal in patients with acute major bleeding. When compared with plasma, this 4 factor PCC product was found to be non-inferior at achieving hemostasis at 24 hours (72.4% vs. 65.4%) and superior at achieving rapid correction of INR to 1.3 or less at 30 minutes (62.2% vs. 9.6%). The recommended dosing strategy for this product is 25–50 units/kg based on patient weight and baseline INR [15]. The fixed dosing used in our patients may have contributed to the results of fewer patients achieving the goal INR of 1.5 or less.

A recent evaluation of PCC3 found suboptimal reversal of warfarin in patients with an INR greater than 5. The INR was reversed to less than 3 in 50% of patients receiving PCC3 25 units/kg and 43% of patients receiving PCC 50 units/kg. Transfusion of additional FFP (mean of 2.1) was required to provide further INR lowering to below 3, resulting in 89% and 88% of patients in the 25 U/kg and 50 U/kg groups achieving that INR goal, respectively [16]. Imberti et al. used a PCC3 administered at 35–50 units/kg in patients with ICH effectively reversed the INR from a mean of 3.5 (range 2.0–9.0) to 1.3 (range 0.9-3.0) with 75% of patients achieving an INR of less than 1.5 within 30 minutes of PCC3 administration. These authors also noted achieving an INR less than 1.5 within 30 minutes fewer in patients whose INR was 4–6 (33%) compared to those whose INR was 2.0-3.9 (89%) [17]. These results led some to suggest that PCC3 use be limited to patients whose INR is 4 or less until further data on PCC3 use in higher INR levels is available [18].

Recombinant factor VII, when complexed with tissue factor, accelerates the extrinsic clotting cascade to promote coagulation. Several reports, mostly in patients who have suffered acute intracranial hemorrhage secondary to warfarin anticoagulation, reported rFVIIa dosed at 10–100 mcg/kg or 1200–9600 mcg to rapidly and completely reversed the INR [5,19-22]. In a study evaluating lower doses of rFVIIa for warfarin reversal, Dager et al. reported that 16 patients who received 1200 mcg of rFVIIa effectively achieved reversal of the INR a mean INR of 2.8 to 1.07 in a mean time of 35 minutes [13].

Our results show that both PCC3 and LDrFVIIa reverse warfarin anticoagulation, but that LDrFVIIa was more predictable at complete reversal of the INR. We used Profilnine® SD, PCC3 containing not more than 35 I.U. of factor VII per 100 units of factor IX [23]. The lower amounts of factor VII may have resulted in smaller reductions in the INR which is highly sensitive to inhibition by factor VII and may not have reflected its true effect on the coagulation system. In contrast, LDrFVIIa rapidly and completely reversed the INR in our patients.

Whether this is due to the sensitivity of the INR to factor VII activity or whether it reflects the true effect on the coagulation system can only by inferred from our data in that there were no cases of unexpected bleeding in either group. Skolnick et al. provided data that questions whether the INR is the most accurate test to measure the true anticoagulation reversal effects of coagulation factors and the ability of rFVIIa to completely reverse warfarin anticoagulation. In a study evaluating the effects of rFVIIa on coagulation parameters and bleeding from punch biopsies in 85 study subjects anticoagulated with warfarin (INR was 2.5 ± 0.3). Subjects underwent biopsies at 4 time points: 1) prior to warfarin anticoagulation; 2) after an INR of 2.5 or greater was achieved; 3) 13 minutes after receiving an injection of placebo or one dose of rFVIIa (administered 2 hours after the second biopsy) as either 5, 10, 20, 40, or 80 mcg/kg; 4) 5 hours after the placebo or rFVIIa dose was administered. Coagulation parameters aPTT, PT, and INR and thrombin generation were collected in all patients at each biopsy. The mean INR was significantly lower in those patients that receiving rFVIIa at all doses (1.2-1.5) when compared to those that receiving placebo (2.5), p < 0.001. However, bleeding duration (min) and blood loss (ml) were not different between those who received placebo and those who received rFVIIa at any dose [24].

To compare the effects of rFVIIa and PCC on anticoagulation reversal, Dickneite administered saline, 100 mcg/kg rFVIIa, or PCC 50 units/kg (Beriplex® P/N-a 4 factor PCC) in rats anticoagulated with either one dose of 2.5 mg/kg phenprocoumon (acute model) or two doses of phenprocoumon dosed 24 hours apart (sustained model). Anticoagulation was reversed 16 hours after the single dose model or 48 hours after the 2 dose model. Both rFVIIa and PCC4 were effective at lowering the PT compared to placebo. However, in the sustained model, PCC4 was significantly more effective at reducing blood loss compared to placebo and rFVIIa [25]. The author suggests the difference in the results are due to the low levels of other clotting factors, aside from factor VII, in rFVIIa compared to this PCC4 product. In the 9th edition of the American College of Chest Physicians Evidence Based Clinical Practice Guidelines on the Pharmacology and Management of Vitamin K Antagonists released in February 2012, a specific recommendation was made to prefer four-factor PCC over FFP for rapid reversal of anticoagulation in VKA-associated major bleeding [10]. Due to limited evidence supporting rFVIIa, the guidelines also state that rFVIIa cannot be recommended unless other more effective agents are not available in the setting of life threatening bleeding [3].

The administration of coagulation factors is associated with thromboembolic events. In our study groups, the incidence of thromboembolic events was equal in both groups. Safaoui et al. reported no thromboembolic events in 28 patients receiving 2000 units of PCC3 (Konyne™ or Profilnine™) [26]. In a recent case report a dose of 50 units/kg of PCC for warfarin reversal was associated with fatal intracardiac thrombosis in a patient who had also received 24 micrograms of desmopressin for suspected uremic platelet dysfunction and fifty minutes later underwent pericardiocentesis [27]. There is more literature addressing the risk of thromboembolic events associated with rFVIIa. A recent publication evaluated 35 randomized clinical trials involving 4468 patients. A total of 498 thromboembolic events were reported (11.1%). Arterial thrombembolic events were higher in those that received rFVIIa (5.5% rFVIIa vs. 3.2% Placebo, p = 0.003), particularly coronary events (2.9% vs. 1.1%, p = 0.002). Venous thromboembolic events were not different between rFVIIa and placebo (5.3% rFVIIa vs. 5.7%. placebo) [28]. There were no arterial thromboembolic events in any of the patients in our study groups.

There were several limitations to our study. This was a retrospective, observational study at a single center in which the choice of coagulation factor was at the discretion of the prescriber and INR monitoring was not conducted in accordance to any protocol. While the average time between the pre and post coagulation factor INR was similar in the two groups (3:53[2:32-7:17] PCC3 compared to 4:30[2:21-6:25] LDrFVIIa, p = 0.78), the lack of standardization prevents us from determining the actual timing of INR reversal. It is possible that some patients achieved a goal INR of less than or equal to 1.5 in a significantly shorter time period given the observation that coagulation factor levels would be expected to rise quickly after administration rFVIIa or PCC and a literature review of 4-factor PCC corrected the INR within 10 to 20 minutes of administration [9]. Another limitation of this study is that there was no scheduled or systematic screening for thromboembolic events. Although patients receiving PCC and rFVIIa are generally assessed for signs of thromboembolic complications, events could have gone undetected.

## Conclusions

In patients with serious or life threatening bleeding, low dose activated recombinant factor VII provided a more rapid and complete reversal of warfarin anticoagulation as determined by reduction of the INR to a value of 1.5 or less when compared to 3 factor prothrombin complex concentrate. The effect on systemic coagulation cannot be determined by this study since we did not measure coagulation factor concentrations or bleeding time in correlation with the INR. Thromboembolic events were not different between the groups. LDrFVIIa and PCC3 groups were comparable in terms of cost for reversal therapies. Further research is needed to provide greater information about the impact of coagulation factor concentration changes related to the administration of coagulation factors, the effect these products have on restoring normal coagulation and at different doses, and the true impact of these products on the actual impact of restoring hemostasis.

## Abbreviations

VKA: Vitamin K antagonist; INR: International normalized ratio; ACCP: American College of Chest Physicians; FFP: Fresh frozen plasma; PCC: Prothrombin complex concentrate; PCC3: 3 factor prothrombin complex concentrate rFVIIa, Recombinant Factor VIIa; LDrFVIIa: Low dose recombinant Factor VIIa; DVT: Deep vein thrombosis; CVA: Cerebrovascular accident; IQR: Interquartile range.

## Competing interests

None of the authors have any conflicts of interest or special declarations to make regarding the contents of this manuscript.

## Authors’ contributions

SC contributed to the study idea, collecting and statistical analysis of data, and preparation of the manuscript. EI contributed to the study idea and preparation of the manuscript. NA-K contributed to data collection and statistical analysis and manuscript preparation. NR contributed to data collection and manuscript preparation. KH contributed to data collection and manuscript preparation. JV contributed to the concept of the study and critical review of the manuscript. RR contributed to the concept of the study and critical review of the manuscript. All authors read and approved the final manuscript.
